# Supplementary material for: Sustainable Recovery of Valuable Nanoporous Materials from High-Chlorine MSWI Fly Ash by Ultrasound with Organic Acids
Source: Molecules. 2022 Mar 31;27(7):2289. doi: 10.3390/molecules27072289 (PMC9000401; doi:10.3390/molecules27072289)
Supplement: Supplementary file 1 [file molecules-27-02289-s001.zip › molecules-1651506-supplementary.pdf]

Supplement Data

Sustainable recovery of valuable nanoporous materials from high-chlorine MSWI fly ash by ultrasound with organic acids

Thanh Tam Nguyen[1,2*], Cheng-Kuo Tsai[3], Jao-Jia Horng[3*]

1. Faculty of Environment, University of Science (VNUHCM), Ho Chi Minh City 700000, Vietnam.
2. Vietnam National University Ho Chi Minh City, Ho Chi Minh City 700000, Vietnam.
3. Department of Safety, Health, and Environmental Engineering, National Yunlin University of Science and Technology, Yunlin 64002, Taiwan.

Table S1. ANOVA for Cl removal performance of Citric acid

Cl removal performance of Citric Acid – Analysis of variance				
Source	Degree of freedom	Sum of Squares	F	Percentage Contribution (%)
A	3	7.328	0.80	3.342
B	3	8.557	0.94	3.902
C	3	200.88	22.06	91.61
D	3	2.493	0.27	1.136

Table S2. ANOVA for Cl removal performance of Ascorbic acid

Cl removal performance of Ascorbic Acid – Analysis of variance				
Source	Degree of freedom	Sum of Squares	F	Percentage Contribution (%)
A	3	2.646	1.06	2.205
B	3	5.454	2.18	4.546
C	3	109.27	43.58	91.07
D	3	2.608	1.04	2.173

Table S3. ANOVA for Cl removal performance of EDTA-Na_2

Cl removal performance of EDTA-Na_2 – Analysis of variance				
Source	Degree of freedom	Sum of Squares	F	Percentages Contribution (%)
A	3	0.449	0.68	2.564
B	3	0.472	0.72	2.694
C	3	16.24	24.73	92.73
D	3	0.352	0.54	2.009

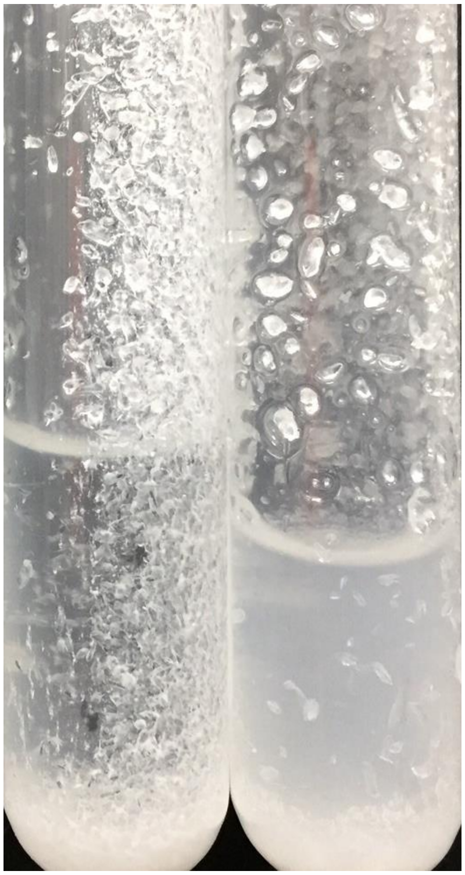

Figure S1. Crystallization of leachate
